# Monocytes Loaded with Indocyanine Green as Active Homing Contrast Agents Permit Optical Differentiation of Infectious and Non-Infectious Inflammation

**DOI:** 10.1371/journal.pone.0081430

**Published:** 2013-11-25

**Authors:** Joani M. Christensen, Gabriel A. Brat, Kristine E. Johnson, Yongping Chen, Kate J. Buretta, Damon S. Cooney, Gerald Brandacher, W. P. Andrew Lee, Xingde Li, Justin M. Sacks

**Affiliations:** 1 Department of Plastic and Reconstructive Surgery, Johns Hopkins University School of Medicine, Baltimore, Maryland, United States of America; 2 Division of Infectious Diseases, Department of Medicine, Johns Hopkins Bayview Medical Center, Baltimore, Maryland, United States of America; 3 Department of Biomedical Engineering, Johns Hopkins University School of Medicine, Baltimore, Maryland, United States of America; Glasgow University, United Kingdom

## Abstract

Distinguishing cutaneous infection from sterile inflammation is a diagnostic challenge and currently relies upon subjective interpretation of clinical parameters, microbiological data, and nonspecific imaging. Assessing characteristic variations in leukocytic infiltration may provide more specific information. In this study, we demonstrate that homing of systemically administered monocytes tagged using indocyanine green (ICG), an FDA-approved near infrared dye, may be assessed non-invasively using clinically-applicable laser angiography systems to investigate cutaneous inflammatory processes. RAW 264.7 mouse monocytes co-incubated with ICG fluoresce brightly in the near infrared range. In vitro, the loaded cells retained the ability to chemotax toward monocyte chemotactic protein-1. Following intravascular injection of loaded cells into BALB/c mice with induced sterile inflammation (Complete Freund’s Adjuvant inoculation) or infection (Group A Streptococcus inoculation) of the hind limb, non-invasive whole animal imaging revealed local fluorescence at the inoculation site. There was significantly higher fluorescence of the inoculation site in the infection model than in the inflammation model as early as 2 hours after injection (p<0.05). Microscopic examination of bacterial inoculation site tissue revealed points of near infrared fluorescence, suggesting the presence of ICG-loaded cells. Development of a non-invasive technique to rapidly image inflammatory states without radiation may lead to new tools to distinguish infectious conditions from sterile inflammatory conditions at the bedside.

## Introduction

In 2004, there were 869,777 hospital admissions in the United States for skin or soft tissue infections [1]. Incorrect diagnosis of these infections can lead to unnecessary antibiotic use, greater healthcare costs and may result in higher rates of re-admission [2]. Skin inflammation is a common pathophysiologic process characterized by clinical signs including erythema, edema, tenderness, and warmth. Despite this overarching phenotype, its varied etiologies include both sterile and infectious processes. Different, often contradictory, treatments are required depending upon the underlying cause. Cellulitis, the most common cause of infectious cutaneous inflammation, is easily mistaken for inflammation secondary to trauma, dermatitis, or non-infectious inflammation [2]. Therefore, a rapid mechanism for accurately imaging and diagnosing the etiology of inflammation would be of considerable clinical utility.

To date, an objective, non-invasive and reproducible method to characterize and differentiate states of cutaneous inflammation by underlying etiology does not exist. Current methodologies include interpretation of indirect and non-specific laboratory testing, clinical appearance of the skin, and results of available wound biopsy or culture. Non-invasive imaging modalities such as computed tomography, magnetic resonance imaging, and ultrasound can fail to distinguish infection without significant abscess. Dedicated studies, such as tagged white blood cell scans, involve ionizing radiation, and are not specific. Some methods are available in the laboratory, but involve genetic alteration or are cytotoxic, such as bioluminescence or quantum dots, or use non-FDA approved fluorophores or nanoconstructs, all of which preclude clinical translation. Others such as two-photon and multiphoton microscopy are severely limited by depth of penetration and size of the field of view. Biopsy and culture remain the gold standards for assessing characteristic variations in leukocytic infiltration and bacteriology. However, these modalities are temporally inefficient and may lead to diagnostic delays--a particular concern in immunosuppressed patients.

Optical imaging could be used to non-invasively assess local cellular infiltrate. Near infrared (NIR) imaging in the wavelengths of 700-900 nm provides deep cutaneous penetration because it is subject to less scattering and autofluorescence from biological tissues [3]. Clinical imaging systems designed for NIR angiography are commercially available, with multiple applications in oncology, vascular and reconstructive surgery, and wound healing. 

Indocyanine green (ICG) is a relatively inexpensive, safe, tricarbocyanine NIR dye, which has been approved by the FDA for over 50 years, with common application in ophthalmic angiography, cardiology, and hepatology [4–7]. Previous research has shown the potential to load cells with ICG in vitro, yielding cells that fluoresce in the NIR range. Human embryonic stem cells and human embryonic stem cell-derived cardiomyocytes [8] and isolated human monocytes and spleen macrophage cell lines [9] have been loaded with ICG in vitro. Building on this literature, and with an eye toward future clinical application, we developed a rapid and scalable approach to loading mouse-derived monocytes with ICG. We validated this technique in murine models of sterile and infectious inflammation. After injection, we used a clinically available NIR imaging system for whole animal imaging. The aim of this study was to show that this technique is capable of imaging inflammation in sterile and infectious conditions. Results from the study indicate that there is a different fluorescence pattern for sterile versus infectious inflammation. 

## Results

### ICG Cell Loading

**Figure 1:**
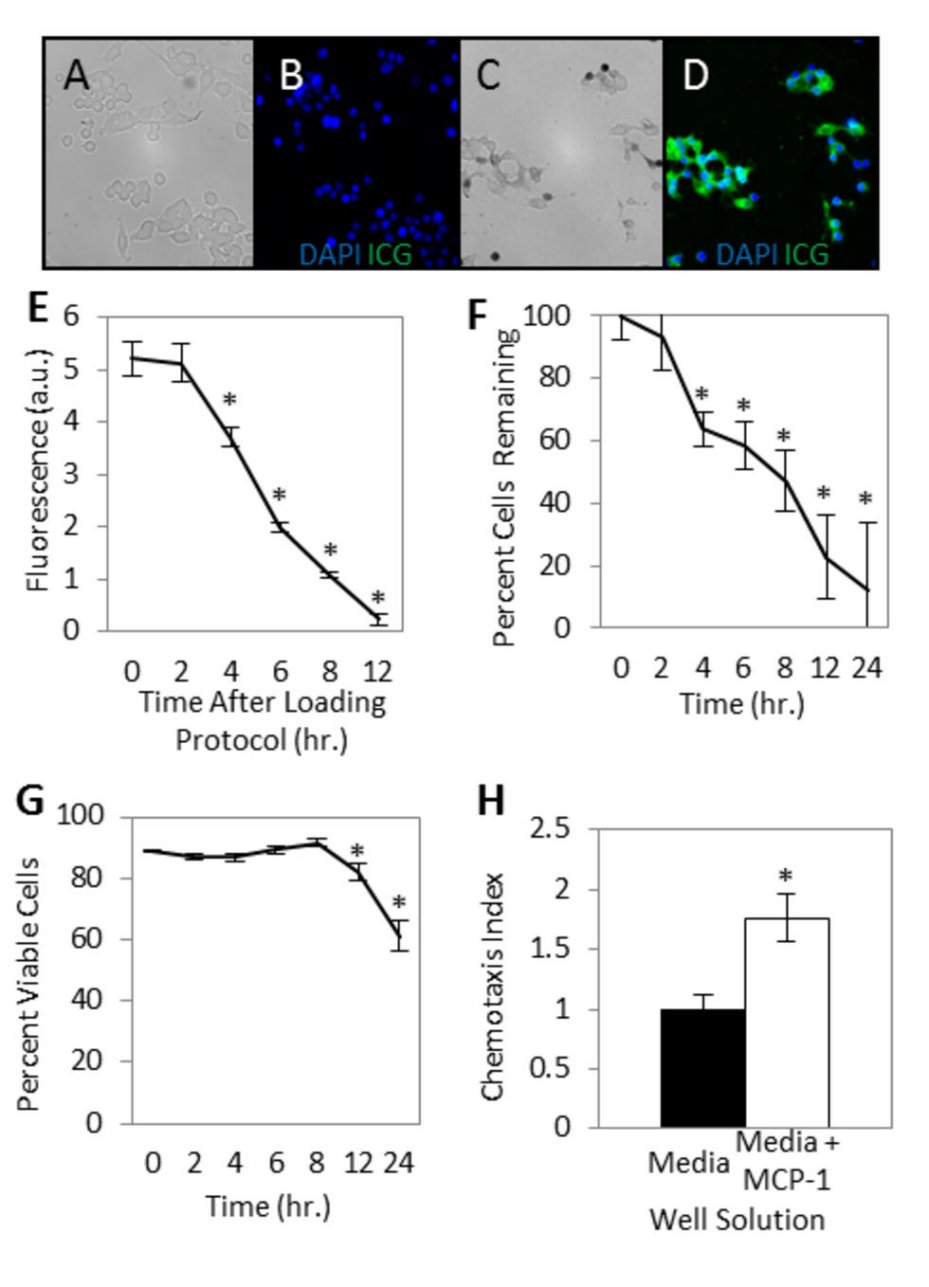
In vitro studies of ICG loaded monocytes. (*A*, *B*) Representative images, RAW 264.7 mouse monocytes incubated in media alone and stained with DAPI. (*A*) Brightfield image. (*B*) DAPI blue nuclear staining and no NIR fluorescence (pseudocolor green). (*C*, *D*) Representative images, Monocytes after incubation in media with ICG solution and stained with DAPI. (*C*) Brightfield image. (*D*) DAPI fluorescence (blue) and NIR ICG fluorescence (pseudocolor green). (*E*) *Ex* *vivo* cellular fluorescence over time. Monocytes incubated in media with ICG display an average NIR fluorescence of 5.22±0.34 arbitrary units (a.u.) above background (control non-ICG loaded cells) following the loading procedure, decreasing toward background over the following 12 hours (N=10). (*F*, *G*) Yield and viability of monocytes after ICG loading procedure. (*F*) Cell yield, after maintaining the cells *ex* *vivo* in culture media and washing and centrifuging at each time point, decreases over the first 24 hours after loading with ICG. (*G*) Viability of cells remains greater than 80% for more than 12 hours (N=10). (*H*) ICG loaded monocyte chemotactic capacity. Chemotactic index is the number of cells migrating through the chemotaxis filter toward media with the chemoattractant MCP-1 relative to the number of cells migrating through the chemotaxis filter toward media alone. The average number of migrated ICG-loaded monocytes per five high powered fields was 86.0±27.8 with MCP-1 in the bottom well, and 48.9±16.5 with chemotaxis media alone in the bottom well (N=3). All error bars represent SEM. *P<0.05.

The cell loading efficiency of ICG depends on many parameters including dye concentration, carrier solution, incubation conditions, and washing protocol. Cells loaded at the optimal ICG concentration displayed high NIR fluorescence intensities (5.22±0.34a.u.) above background (non-loaded cells) (Figure 1A-E). When the cells were subsequently incubated in culture media at 37°C, and washed and centrifuged at each successive time point, fluorescence gradually decreased, returning to baseline in these conditions after 12 hours. Cell counts after centrifugation revealed a progressive decrease in the yield, or number of cells present, over time after the loading procedure (Figure 1F). Despite the decrease in total yield of cells, cell viability (assessed by trypan blue exclusion) remained greater than 80% up to 12 hours after the loading procedure, decreasing to 61.2±5.2% at the 24 hour time point (Figure 1G). 

### In Vitro Chemotaxis

To assess maintenance of cellular function following the loading protocol, an in vitro chemotaxis assay was performed. Monocyte chemotactic protein-1 (MCP-1) was chosen as the chemoattractant in the microplate chemotaxis assay. The ability of the monocytes to chemotax and traverse the 5μm filter pores remained above baseline (*P*<0.05) after the loading procedure (Figure 1H). The average number of migrated cells per five randomly selected high power fields (HPF; 400x) was (48.9± 5.8) with chemotaxis media alone in the bottom well and (86.0± 9.8 per HPF) with chemotaxis media and MCP-1 in the bottom well. The ICG-loaded cells had a chemotactic index of 1.8± 0.2 relative to the random motion of the cells in wells above chemotaxis media alone (chemotactic index 1.0). 

### In vivo Imaging

#### Injection of ICG-loaded monocytes

**Figure 2:**
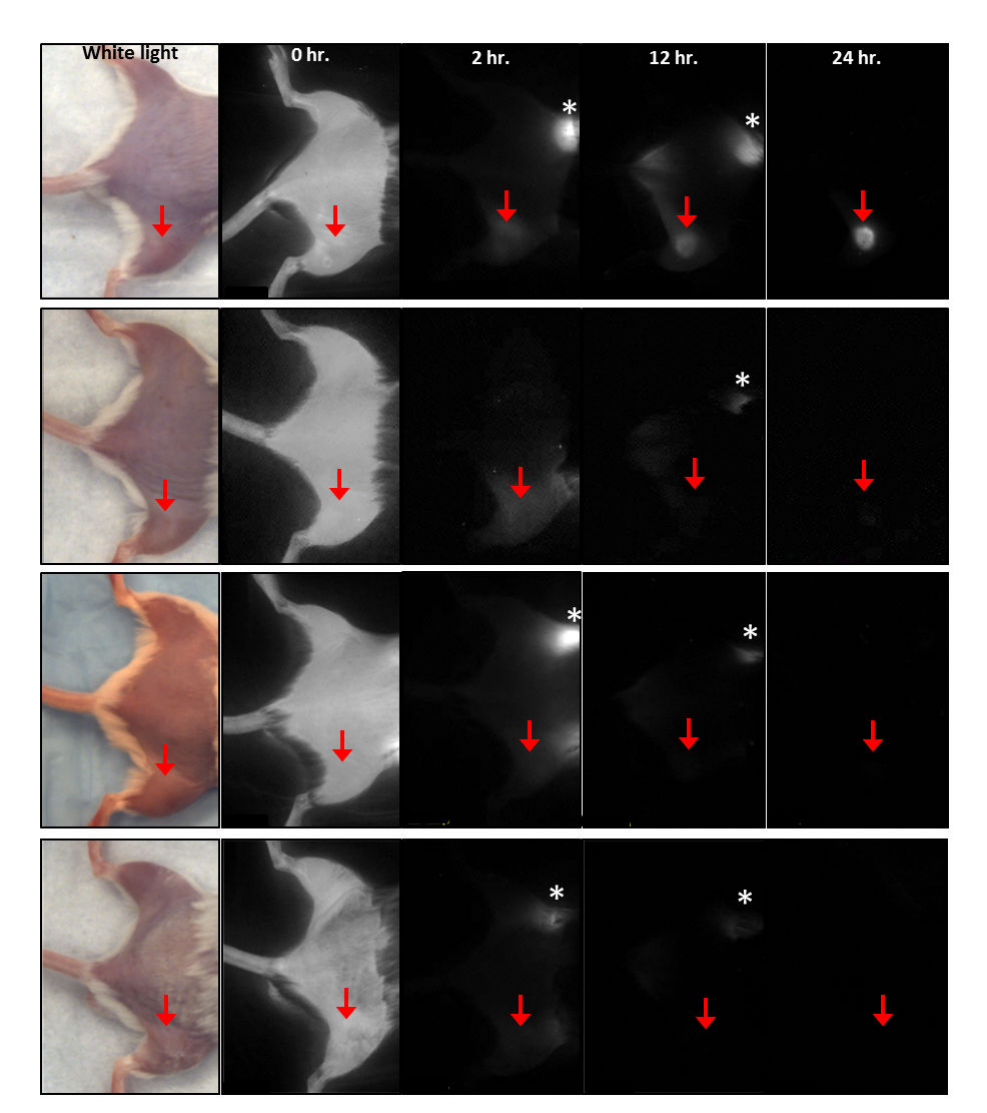
Near infrared images taken after systemic injection of ICG-loaded monocytes or ICG solution. Top row: Infection model, cellular injection. Second row: Infection model, solution injection. Third row: Inflammation model, cellular injection. Fourth row: Inflammation model, solution injection. Red arrow indicates inoculation site with Complete Freund’s adjuvant in the inflammation model, or Group A Streptococcus in the infection model. Asterisk indicates ICG being excreted through the bowel.

**Figure 3:**
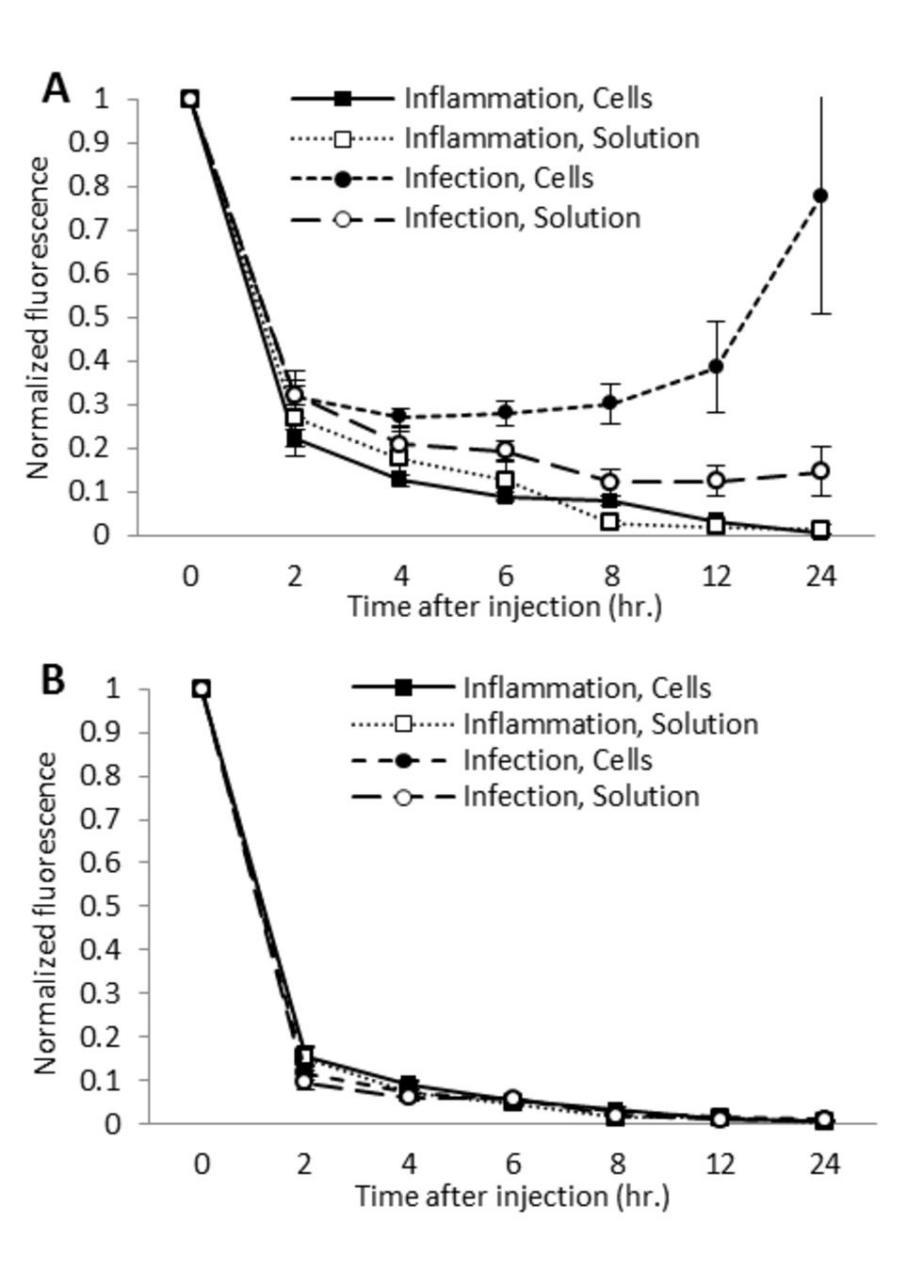
Normalized fluorescence for in vivo imaging of ICG labeled cells and ICG solution. (*A*) Normalized fluorescence of inoculation area. Normalized fluorescence is significantly higher in the infection model (N=8) than in the inflammation model (N=8) as early as 2 hours after cellular injection (p<0.05). After injection of ICG solution, normalized fluorescence in the infection model (N=8) is significantly higher than in the inflammation model (N=8) at the 8, 12, and 24 hour time points (p<0.05). Normalized fluorescence after injection of cells was significantly higher than after injection of ICG solution from the 6 hour time point onward (p<0.05). Fluorescence intensity was normalized to the intensity at 0 hours after injection. (*B*) Normalized fluorescence of contralateral control area. Normalized fluorescence generally decreases throughout the study in all groups (N=8 each). All error bars represent SEM.

Whole body NIR imaging immediately following systemic injection of ICG-loaded cells revealed diffuse fluorescence in both infection (subcutaneous *Streptococcus pyogenes* inoculation) and inflammation (subcutaneous complete Freund’s adjuvant (CFA) inoculation) model mice, as cells disseminated throughout the vasculature (Figure 2). The fluorescence of the inoculation area and the contralateral control area were normalized to their florescence intensity at 0 hours. As time from the injection of loaded cells increased, background fluorescence intensity of the control limbs decreased (Figure 3B). The normalized fluorescence in the infection model inoculation area decreased to a nadir of 0.272±0.018 (27.2% of the original fluorescence) at 4 hours, before increasing to 0.777±0.27 (77.7%) at 24 hours (Figure 3A). The contralateral limb normalized fluorescence decreased to 0.074±0.007 (7.4%) by 4 hours and 0.009±0.001 (0.9%) by 24 hours (Figure 3B). In the sterile inflammation model, the normalized fluorescence of the inoculation area decreased throughout the experiment, with a normalized fluorescence of 0.13±0.01 (13%) at 4 hours and 0.004±0.001 (0.4%) at 24 hours (Figure 3A). Normalized fluorescence in the contralateral control limbs of these animals also decreased consistently, 0.090±0.009 (9.0%) at 4 hours and 0.004±0.001 (0.4%) at 24 hours (Figure 3B). Normalized fluorescence in the infection area was significantly higher than that in the sterile inflammation area from the 6 hour time point onward (p<0.05). 

#### Injection of ICG solution

Immediately following the systemic injection of ICG solution or ICG-loaded monocytes, the fluorescence levels of the contralateral control limbs, 78.8±16.7 a.u. and 78.7±14.1, respectively, were similar. Following ICG solution injection, normalized fluorescence in the experimental limb in the infection model decreased to 0.122±0.031 (12.2% of the fluorescence at 0 hours) at 8 hours and remained relatively stable, while the sterile inflammation inoculation area decreased to 0.028±0.008 (2.8% of the fluorescence at 0 hours) at 8 hours and 0.014±0.012 (1.4%) at 24 hours (Figure 3A). The infection inoculation area displayed a significantly higher normalized fluorescence than the sterile inflammation inoculation area at the 8, 12, and 24 hour time points after ICG solution injection (p<0.05) (Figure 3A). In the contralateral control limbs, normalized fluorescence decreased to 0.019±0.003 (1.9%) and 0.015±0.003 (1.5%) at the 8 hour time point in the infection and sterile inflammation models, respectively, decreasing to 0.008±0.002 (0.8%) and 0.003±0.001(0.3%) at 24 hours (Figure 3B). 

When comparing the injection of ICG-loaded cells to injection of ICG solution, injection of cells in the infection model led to significantly higher normalized fluorescence at the 6 8 hour time point after injection (0.30 vs. 0.12; 30% of the original fluorescence versus 12% of the original fluorescence) and at all subsequent time points (P<0.05), indicating that injection of loaded cells allowed superior visibility of local infection. 

### Histopathology

**Figure 4:**
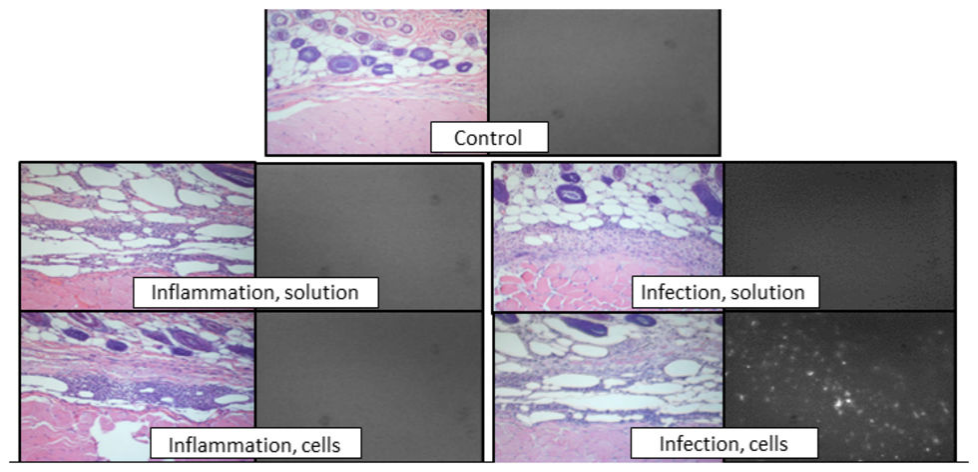
Punctuate fluorescence at the inoculation site of animals receiving injection of ICG-loaded cells. *Left* *image*: representative paraffin-embedded sections from inoculation site or control limb stained with hematoxylin and eosin and imaged using light microscopy. *Right* *image*: representative frozen sections imaged with a near infrared filter.

Representative sections of cryopreserved tissue harvested from animals in the inflammation and infection groups 24 hours after injection of either ICG-loaded cells or ICG solution were examined. Tissue from animals receiving ICG solution displayed a relatively low, homogenous level of fluorescence. In animals receiving ICG-loaded cell injections, punctate areas of bright fluorescence (points) were seen in the bacterial inoculation area but not the sterile inflammation inoculation area. Minimal to no heterogeneous background fluorescence was observed in the control limbs of both models (Figure 4).

## Discussion

There is no single clinically effective test to distinguish sterile inflamed tissue from infectious cellulitis. Further, assessment of the inflammatory infiltrate and its natural evolution could suggest pathogenetic mechanisms. Although laboratory techniques are currently available to track cells in vivo in animal models, they are not clinically applicable due to toxicity (radiolabelling, quantum dots) or need for genetic manipulation (bioluminescence). Other investigators have proposed using fluorescence imaging to visualize inflammation. Rodent leukocytes and macrophages have been labeled with non-clinically approved dyes and injected systemically to image induced inflammation in animal models [10–12]. Despite toxicity, radiolabelling currently provides the most clinically relevant option. ICG is already in common use clinically, and thus paves the pathway towards clinical translation. NIR imaging using inflammatory cell populations loaded with ICG potentially advances and improves upon current clinical techniques to image infectious and sterile inflammation.

In this animal study, we developed a technique that shares a number of similarities with radiolabeled cell imaging, but, notably, without the toxic effects. Also in contrast, our strategy provides live imaging using cellular chemotaxis and offers clear differentiation of a sterile inflammation model and a bacterial infection model. We have shown that cultured mouse monocyte-macrophages can be loaded with ICG and display high levels of NIR fluorescence (Figure 1E). High fluorescence combined with high viability (Figure 1F, 1G) and retained chemotaxis (Figure 1H) illustrate the promise of such loaded cells for in vivo cell homing studies. When cells were injected systemically for whole animal non-invasive imaging with a NIR optical system, sites of sterile inflammation and infection were highlighted compared to the contralateral background site (Figure 2). In addition, normalized fluorescence in bacterial inoculation areas was higher than in sterile inflammation inoculation areas as early as 6 hours after cellular injection, with the difference becoming more pronounced up to 24 hours after injection (78% vs. 0.4% of the fluorescence at t0, respectively) (Figure 3A). This suggests that monocyte/macrophages may be more involved in response to infection as compared to sterile inflammation. 

Isolation of monocytes and macrophages, ex vivo cellular manipulation, and autotransfusion is used clinically in adoptive cellular immunotherapy [13–16], and for imaging possible infection in tagged white blood cell scans. Given the current clinical applications of ICG and cell autotransfusion, our techniques could lead to a translational imaging strategy for visualizing inflammatory states by underlying etiology. If applied at the bedside, the ability to detect an influx of inflammatory cells could substantially reduce time to accurate diagnosis and effective treatment compared to delays associated with other methods such as culture, biopsy, or tagged white cell scans. 

In addition to future applications as a clinical diagnostic device, this approach may serve as a powerful research tool to better define pathogenesis (alterations in lymphocyte migration and soft tissue perfusion) according to cutaneous and soft tissue inflammatory states. This is of particular significance because although a common condition, descriptions of the nature of cellular and soluble mediators of inflammation, foci of bacterial growth and alteration in perfusion in cellulitis are lacking. The greater normalized fluorescence after ICG-labeled cell injection and ICG solution injection in animals with infection compared to animals with sterile inflammation (Figure 3A) could indicate that monocyte-macrophage migration to the local area is more characteristic of infection, and that infection may be associated with significant alterations in perfusion, including capillary and lymphatic vessel dysfunction. 

 Our studies reveal excellent viability for the ICG-loaded monocytes (Figure 1G), confirming the low toxicity of ICG, and safety of the preparation procedure for the monocytes. These results are consistent with other reports of ICG cellular labeling, including in vitro loading of human embryonic stem cells and human embryonic stem cell-derived cardiomyocytes [8], and human blood monocyte and spleen macrophage cell lines [9]. Although our in vitro studies involving incubation in culture media and successive washing and centrifuging at every time point, meaning 6 wash cycles by 24 hours, showed relatively rapid decay of fluorescence (Figure 1E), in vivo imaging suggested longer persistence of fluorescence of cells in the biological milieu with appropriate chemokine and molecular signals. 

NIR imaging after intravenous injection of ICG solution has been reported to image inflammation such as arthritis [17,18]. Injection of ICG solution allows visualization of perfusion, so hyperperfused areas, such as inflamed tissue, display increased fluorescence. In light of this potential application, we performed imaging after injection of ICG solution in the sterile inflammation and infection models to determine if cellular injection led to more specific, differential fluorescence compared to injection of free (not cell-loaded) ICG solution. Our results agreed with the existing literature, that the inflammatory focus could be visualized (Figure 3A). Fluorescence of the inoculation area decreased more rapidly than after cellular injection, but showed elevated fluorescence for longer than expected, given the typical plasma half-life of ICG solution of a few minutes [18]. Prolonged washout of the inoculation area could indicate microcirculatory disturbances, such as capillary leakage and lymphatic dysfunction. Moreover, injection of cells led to a greater differential in the fluorescence levels of the sterile inflammation and infection models from the 8 hour time point onward when compared to injection of free ICG solution (Figure 3A). Such findings indicate that fluorescence at the inoculation area after cellular injection was not solely due to the increase in blood flow to the inoculation area. 

While the injection contained 2 million cells, upon histological examination, far fewer cells appeared to infiltrate the inoculation area (Figure 4). After initial trafficking to the lung, liver, and spleen, radiolabeled macrophages have been shown to accumulate in an area of paw inflammation [19]. Although the radioactivity at the inflammation site represented only 0.2% of the originally administered dose, the radioactivity was specific [19]. Similarly, although most cells are lost after administration, the smaller number remaining is relatively specifically located at the site of inflammation, allowing visibility on whole animal imaging. 

Although the monocyte-macrophages allowed visualization of both local sterile inflammation and infection and showed there may be potential to differentiate the two conditions, differences in fluorescence could be due to severity of inflammation or timing of imaging within the inflammatory response and not solely due to differing etiology. Inoculation with CFA has been described as a model of local inflammation in rodents [20–22], as has inoculation with bacteria as a model of cutaneous and subcutaneous local infection [23–25]. Animals in all groups shared a similar clinical appearance; however, direct comparison of the degree of inflammation and cellular influx induced has not been performed to date. More work is necessary to elucidate the role of inflammation severity and etiology in the imaging results. 

In addition, greater differences in ICG signal between the models may be seen with injection of a different ICG-loaded cell population. Any isolatable cell population potentially could be loaded with fluorophore for tracking studies. Phagocytic cells, such as macrophages, are more likely to take up ICG during standard incubation, making those cell populations preferential. For example, ICG-loaded neutrophils might provide specific imaging of acute infection. 

This study demonstrates the feasibility of using clinical NIR imaging systems combined with injection of ICG-loaded monocytes for imaging sterile and infectious inflammation in vivo. In vitro studies to assess the feasibility of fluorescently labeling isolated cell populations from human peripheral blood, as well as data regarding the long-term persistence and fate of the injected cells, which are outside the scope of this study, are needed to move toward clinical translation of this methodology. 

## Methods and Materials

### Ethics Statement

This animal protocol was approved by the Johns Hopkins Animal Care and Use Committee (Protocol # MO11M52).

### Materials

ICG (IC-Green, Akorn Inc, Lake Forest, IL) provided as a gift by Dr. Robert Flower, Department of Ophthalmology, University of Maryland School of Medicine, Baltimore, MD. RAW 264.7 cells provided as a gift by Dr. Kristy Weber, Department of Orthopedic Surgery, Johns Hopkins University School of Medicine, Baltimore, MD [26]. MCP-1, penicillin, streptomycin, non-essential amino acids, fetal bovine serum (FBS), Dulbecco’s Modified Eagle’s Medium (DMEM), CFA and phosphate buffered saline (PBS) were purchased from Sigma-Aldrich (St. Louis, MO). *Streptococcus pyogenes* (ATCC 11434) was obtained from American Type Culture Collection (Manassas, VA). 

### ICG Cell Loading

#### Cell culture

The murine monocyte/macrophage cell line RAW264.7, at an unspecified passage number, was maintained in DMEM supplemented with penicillin, streptomycin, non-essential amino acids, and 10% (v/v) FBS. Cells were maintained at 37°C in 5% CO_2_, and subcultured by scraping from the culture flask. 

#### Cell loading

Attached cells were washed 3 times with PBS. ICG powder was reconstituted with sterile, deionized water at a concentration of 25mg/mL. ICG solution was added to PBS in the culture flask and the cells incubated for at 37°C in 5% CO_2_ for 1 hour. Cells were washed several times with PBS and detached from the flask with a cell scraper. Harvested cells were washed with PBS, centrifuging at 1500 rpm. Exclusion of trypan blue from cells was a marker of cell viability. To assess viability over time, loaded cells were suspended in media and incubated at 37°C between time points. At 2, 4, 6, 8, 12, and 24 hours after the loading protocol, cells were washed, centrifuged, and counted using a hemocytometer with trypan blue staining. 

#### Fluorescence imaging

Following the cell loading protocol, cell fluorescence was confirmed using a Zeiss AxioPlan 2 microscope (Carl Zeiss, Thornwood, NY) and 49030 ET single band filter with a 710-760 nm excitation window and 810-875 nm emission window (Chroma Technology Corp, Bellows Falls, VA) and an Opteon black and white camera (Opteon Corp., Cambridge MA). Consistent illumination and acquisition parameters were used. Images of 5 random HPF were recorded for 10 batches of loaded cells immediately following the loading procedure. Fluorescence levels were determined using ImageJ (National Institutes of Health, USA). Imaging was repeated after each count and assessment of cell viability, at 2, 4, 6, 8, 12, and 24 hours after the cell loading protocol. Fluorescence levels were corrected for baseline fluorescence due to ambient light. 

### In vitro Chemotaxis

#### Chemotaxis assay

Aliquots of chemotaxis media (DMEM with 1% (v/v) FBS) or chemotaxis media plus 10 ng/ml MCP-1 were placed in microplate wells (Neuro Probe, Inc., Gaithersburg, MD) and a suspension of ICG-loaded cells (2 x 10^6^ ICG-loaded cells in chemotaxis media) was placed on the 5µm pore filter over each well. Experiments were performed in triplicate. The plate was incubated at 37°C in 5% CO_2_ for 6 hours. Non-migrated cells were scraped off of the filter’s upper surface. The filter was removed and stained with crystal violet. Cells migrating through the filter and remaining attached to the lower side of the filter were counted manually in five random HPF (400 X) for each well. Chemotactic index was calculated as the ratio of cell migration toward media supplemented with MCP-1 divided by cell migration toward media alone. 

### In vivo Imaging

#### Microorganisms

*Streptococcus pyogenes* ATCC 11434 was maintained according to ATCC recommendations, and provided as a 0.5 McFarland suspension in PBS for injection.

#### Mouse models of infection and inflammation

Male BALB/c mice, 8 weeks old, were purchased from Jackson Laboratories (Bar Harbor, ME). Mouse hind limbs were depilated before inoculation. Sterile inflammation was induced in 16 animals using a subcutaneous injection of 100 µl CFA solution to the lateral hind limb. Local infection was induced in 16 mice using a subcutaneous injection of 1 x 10^7^ colony forming units of *S. pyogenes* to the lateral hind limb. The contralateral limb of each mouse served as a control. Experiments were approved by the Johns Hopkins University Animal Care and Use Commitee (ACUC; protocol #MO12M52), and the study was carried out in strict accordance with the recommendations from the Johns Hopkins ACUC. All injections were performed under isoflurane anesthesia or ketamine and xylazine anesthesia, and all efforts were made to minimize suffering.

#### In vivo imaging

10-12 hours after inoculation, when local erythema had developed in the infection and inflammation models, half of the animals in each original group (n=8 each) received an intravenous injection of 2 x 10^6^ ICG-loaded monocytes, while the remaining half (n=8 each) received a systemic injection of 0.25 mg/ml ICG solution. Whole body, NIR imaging was accomplished using a NIR laser angiography system (SPY Elite, LifeCell Corp., Branchburg, NJ) 0, 2, 4, 6, 8, 12, and 24 hours after the injection of cells or solution and under the same conditions. Images were processed using ImageJ software. Fluorescence intensities of the inoculation area and the corresponding area of the contralateral limb were measured and normalized to the fluorescence at 0 hours for each animal. 

### Histopathology

#### Histology

Skin, subcutaneous tissue, and underlying muscle from the area of local fluorescence in the hind limb and the contralateral control hind limb were harvested after euthanasia and preserved in 10% formalin for paraffin embedding and staining with hematoxylin and eosin. Slides were then examined using light microscopy. 

#### Fluorescence microscopy imaging

Additional tissue from the area displaying fluorescence on whole body imaging was embedded in Tissue-Tek O.C.T. Compound (Sakura Finetek, Torrance, CA) for fresh cryosectioning. 8 µm sections were examined microscopically for NIR fluorescence. 

### Statistical analysis

Quantitative results were expressed in (mean ± standard error of the mean) for continuous variables. Differences in group means were tested using two-tailed Student’s T-tests with *p* values less than or equal to 0.05 considered significant. In vivo data was analyzed using analysis of variance (ANOVA) with post hoc multiple comparison (Tukey) test. Statistical analysis was performed using SPSS version 19 software (IBM, Armonk, NY). 

## Conclusion

In animal models of local infection and sterile inflammation, we used whole animal NIR imaging after injection of ICG-loaded monocytes to image inflammation, without radiation, genetic alteration, or biopsy. This initial study indicates that non-invasive in vivo imaging may be used to provide differential fluorescence signals in sterile and infectious inflammatory processes. Given the FDA-approved status of the imaging system and fluorescent dye (ICG) used in this study, there is potential for future clinical translation of this imaging modality. The potential to define the etiology of inflammation efficiently and non-invasively in the clinical setting will allow for more accurate diagnostics and may speed therapeutic interventions. 
