# Correction: Monocytes Loaded with Indocyanine Green as Active Homing Contrast Agents Permit Optical Differentiation of Infectious and Non-Infectious Inflammation

**DOI:** 10.1371/annotation/fb09c61a-a793-4cab-b218-2bfdf384bd96

**Published:** 2014-01-09

**Authors:** Joani M. Christensen, Gabriel A. Brat, Kristine E. Johnson, Yongping Chen, Kate J. Buretta, Damon S. Cooney, Gerald Brandacher, W. P. Andrew Lee, Xingde Li, Justin M. Sacks

The following information was erroneously excluded from the Funding statement: "This work was also supported in part by the National Institutes of Health under grant U54CA151838 through a pilot project."

